# Rapid separation of developing Arabidopsis seeds from siliques for RNA or metabolite analysis

**DOI:** 10.1186/1746-4811-9-9

**Published:** 2013-03-26

**Authors:** Philip David Bates, Jeremy Burke Jewell, John Browse

**Affiliations:** 1 Institute of Biological Chemistry, Washington State University, Pullman, WA 99164, USA; 2 Current address: Department of Chemistry and Biochemistry, The University of Southern Mississippi, Hattiesburg, MS 39406, USA

**Keywords:** Arabidopsis, Seed, Silique, Gene expression, Dissection, Harvest, Metabolic quench

## Abstract

**Background:**

Protein, starch and oil produced in plant seeds are major renewable sources of food, chemicals and biofuels. Developing *Arabidopsis thaliana* seeds are commonly utilized as a model for seed crop research. However, due to the very small size of Arabidopsis seeds efficient collection of large amounts of tissue for gene expression or metabolite analysis is very difficult and time consuming.

**Results/conclusions:**

Here we describe a method that allows very rapid separation and collection of large amounts of developing Arabidopsis seeds from their encapsulating silique tissue after flash freezing whole siliques in liquid nitrogen. The efficient popping open of the frozen siliques on dry ice and filtering the seeds away from the silique tissue with liquid nitrogen cooled funnels and sieves allows large amounts of developing seeds to be quickly isolated while remaining frozen. This method increases the speed of developing seed collection approximately 10 fold over methods which dissect individual siliques one at a time.

## Background

Analysis of gene expression or metabolite levels in different tissues of a biological organism is essential to understanding how different tissues function independently from each other. In plants developing seed tissue is the major source of starch, protein, and oils utilized by humans for food, industrial feedstocks or fuel. Therefore accurate analysis of developing seed metabolism is required to understand how these valuable commodities are produced in plants. In the model plant species *Arabidopsis thaliana*, seeds develop in a non-fleshy fruit tissue known as a silique. The silique tissue has different metabolism and gene expression profiles from the enclosed developing seeds [1,2], and the silique walls contain more tissue on a fresh weight basis than the developing seeds throughout most of silique/seed development. Therefore, to accurately analyze developing seed gene expression or low concentration metabolic intermediates (such as acyl-CoAs) the developing seeds must be removed from the silique. The very small size of developing Arabidopsis siliques and enclosed seeds (>0.5 mm, >20 μg fresh weight per seed) [3] makes rapid collection of adequate amounts of tissue for gene expression or metabolite analysis very difficult. Dissecting Arabidopsis seeds from siliques can take 1-3 min per silique, depending on the skill of the experimentalist. Therefore, collection of large amounts of developing seed tissue can be very time consuming. Here we present a method that allows efficient separation of the frozen and metabolically quenched seeds from silique tissue *after* rapidly flash freezing the siliques in liquid nitrogen. Seeds from many siliques may be collected at the same time greatly reducing the time required to collect large amounts of developing seed tissue.

## Results and discussion

**Figure 1:**
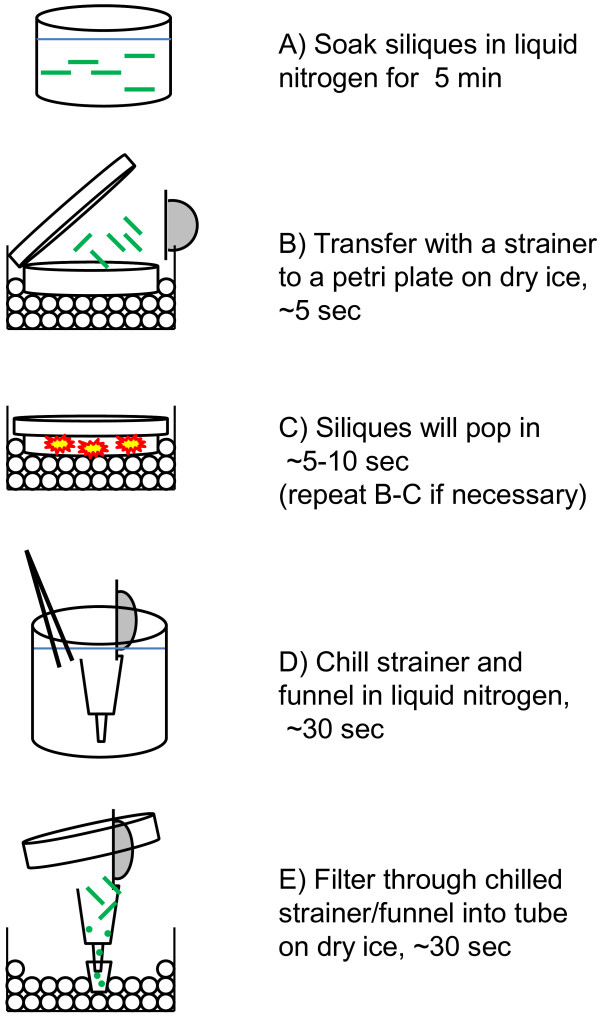
Summarized flow chart of silique popping and seed collection.

The time required to collect all the seeds from 50 developing Arabidopsis siliques by individual silique dissection was approximately 1-2 hrs per person in our hands (~6 individuals, 4 separate attempts). However, our new silique popping method (summarized in Figure 1) allowed us rapidly collect developing seeds from 50 siliques in less than 10 minutes. The silique popping method takes advantage of the fact that developing Arabidopsis siliques soaked in liquid nitrogen will rapidly pop open (like popcorn) when removed from the liquid nitrogen (Additional file 1). The popping efficiently separates the seeds from the silique walls. We believe the popping is caused by liquid nitrogen that soaks into the silique and then rapidly expands to a gas creating the pressure to pop open the silique when the silique temperature is raised above that of the liquid nitrogen. By quickly transferring the siliques from the liquid nitrogen bath to a closed container on dry ice the siliques can be efficiently popped open while seed and silique tissue stays frozen. Subsequent filtering of the popped open siliques through a sieve and funnel that has been chilled with liquid nitrogen into a tube on dry ice allows the seeds to be rapidly separated from the silique tissue while remaining frozen (Figure 1). This silique popping method allows the rapid collection of large amounts of developing seed tissue suitable for downstream applications such as RNA extraction. Popping open of ~190 siliques aged 9-10 days after flowering yielded ~0.8 ml of frozen developing seeds. Three ~0.1-0.2 ml aliquots of developing seed produced 27-38 μg of total RNA each. Our laboratory has successfully utilized this silique popping method to collect sufficient developing seed tissue for gene expression analysis or analysis of very small metabolite pools, such as acyl-CoA, which accumulate at less than 3 pmol/mg fresh weight [4].

**Figure 2:**
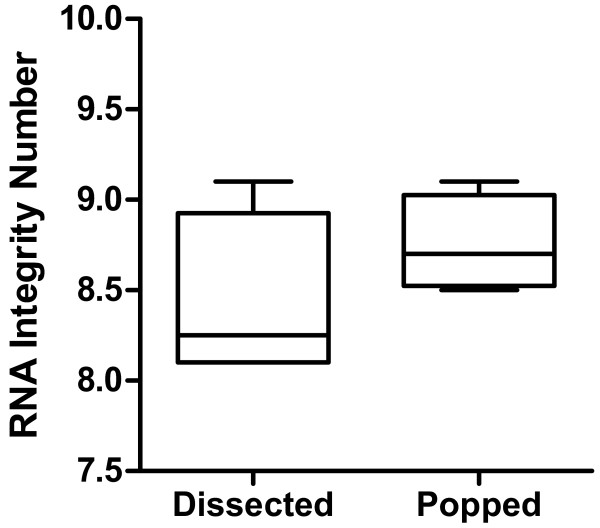
**Comparison of RNA extracted from dissected and popped siliques.** The RNA Integrity Number (RIN) is a measure of RNA degradation to estimate RNA quality for gene expression analysis as measured by an Agilent 2100 bioanalyzer. RIN ranges from 10 (totally intact) to 1 (totally degraded) [11]. A RIN of at least 8 indicates high quality RNA suitable for RNAseq or other analyses. Whisker box plot with four replicates.

**Figure 3:**
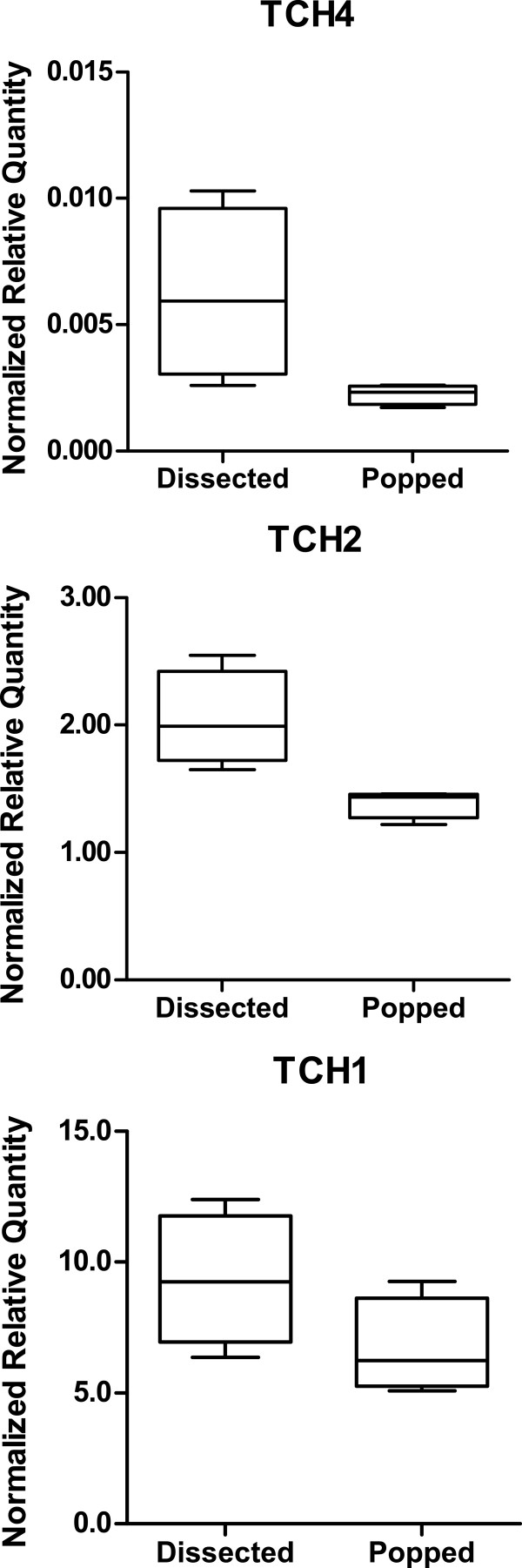
**Comparative gene expression of touch sensitive genes.** qRT-PCR of three genes (TCH4, TCH2, TCH1) that are responsive to handling or touching of plants [6]. Whisker box plot with four replicates.

To test the suitability of seeds collected by the silique popping method for downstream applications such as gene expression analysis, we compared RNA quantity, quality and gene expression between developing seeds collected by dissection or by the silique popping method. Ten developing siliques aged to 9-10 days after flowering were dissected directly into 1.5 ml tubes on dry ice [5]. Total RNA extracted from six replicate samples (each containing 10 dissected siliques) averaged 14.2 ± 1.4 μg each. However, when 50 siliques were popped open together and split into six replicates a higher yield of 22.3 ± 4.4 μg RNA was obtained. This higher yield may be due to more seeds in each replicate from a more complete removal of seeds from each silique by the popping method. RNA extracted from frozen seeds collected by the silique popping method was of a consistently high quality suitable for large scale gene expression analysis by RNAseq (Figure 2). Additionally, expression of Arabidopsis genes known to be up-regulated by the handling or touching of plants [6] had low experimental variability within the samples extracted from seeds collected by the popping method (Figure 3).

## Conclusion

Our silique popping method greatly increases the speed (>10 fold) at which large amounts of developing Arabidopsis seeds can be collected for subsequent analysis of RNA or other low concentration metabolites. Additionally, the rapid quenching of silique/seed metabolism by harvesting the siliques directly into liquid nitrogen ensures minimal changes in metabolite pools due to tissue disruption during dissection.

## Methods

### Silique popping protocol

#### Materials

Liquid nitrogen, a “bed” of dry ice large enough to hold a plastic petri plate, a second dry ice container for 1.5 ml collection tubes, small glass funnel that will fit into 1.5 ml tube, forceps and glass rod, a sieve (or strainer) with pore size large enough for Arabidopsis seeds to fall through (small metal sieves used for straining tea leaves work well).

#### Safety

It is important that all normal safety precautions for using liquid nitrogen and dry ice as indicated by their respective MSDS are observed (http://hazard.com/msds/).

#### Protocol

1. Harvest developing Arabidopsis siliques directly into liquid nitrogen. Let siliques sit in the liquid nitrogen for at least 5 minutes (Figure 1A).

•Take care not to damage the silique tissue. Remove the siliques from the shoot by cutting the pedicel near the stem.

•Siliques ages of ~8-12 days after flowering pop open very well.

2. Put a petri plate on a bed of dry ice and let cool for a few minutes, (while siliques are soaking).

•As long as all materials that come in contact with the seeds/siliques are at dry ice temperature or below then the seeds/siliques will stay frozen through each step.

3. Using a liquid nitrogen cooled metal strainer (or forceps) transfer some frozen siliques from the liquid nitrogen to the petri plate on dry ice and *immediately* put the lid on (Figure 1B).

•Within a few seconds the siliques will start to pop like popcorn (Figure 1C, Additional file 1). The seeds and silique walls that remain in the dry ice cooled dish will remain frozen.

4. Once siliques have popped, remove the lid and repeat until all siliques are the 1.5 ml tube, transferred from the liquid nitrogen bath to the petri plate.

5. Any siliques that do not pop open can be quickly crushed with a liquid-nitrogen-cooled glass rod.

•A thin pair of warm gloves under your lab gloves helps to keep your fingers from freezing while handling liquid nitrogen cooled utensils.

6. Separate the seeds from the silique tissue by filtering through a liquid nitrogen cooled sieve.

a. Place a 1.5 ml collection tube upright in a second container of dry ice.

b. Put sieve into funnel and dip both into liquid nitrogen together with large forceps (Figure 1D) until the liquid nitrogen stops bubbling rigorously (~20-30 seconds).

c. *Immediately* place the cooled sieve/funnel onto the 1.5 ml tube.

d. *Immediately* pour the frozen seeds/siliques from the petri plate into the sieve/funnel and tap a few times. Most of the seeds will flow into the 1.5 ml tube while most of the siliques will be caught in the sieve (Figure 1E).

•A second straining of the collected seeds through the liquid nitrogen cooled sieve will help remove any silique bits that ended up with the seeds.

7. Once collected the seeds may be stored at -80°C until needed for further analysis.

### RNA analysis

Frozen seed samples were ground to a fine power with a bead beater and RNA extracted by [7], DNA was removed by the DNA-Free RNA Kit (Zymo Research). Total RNA was quantified on a nano-spectrophotometer, values reported are average and standard error. qRT-PCR followed standard procedures [8,9] and was normalized to TIP41-like [10]. Primers utilized for RT-PCR: qTCH1-f, ATTTGCATGATTGGTGGAGATATG; qTCH1-r, CCATCGGTTTCAATCCAACTTAC; qTCH2-f, GGAAGATTTCGCCGGAGATTAG; qTCH2-r, AGAGCAGAAGATATAGAAACAACCG; qTCH4-f, TCACAAGAGCTTGACTCAACAG; qTCH4-r, TCTTGTTCCTCTCTCAACTCTTTAC. RNA quality was measured on an Agilent 2100 bioanalyzer [11].

## Competing interests

The authors declare that they have no competing interests.

## Authors’ contributions

PB designed/tested the method and wrote the manuscript. JJ tested the method and analyzed RNA prepared utilizing the method. JB gave critical input for method design and manuscript writing. All authors read and approved the final manuscript.

## Supplementary Material

Additional file 1 **Popping siliques as in protocol step 3 (Figure** **1B,C).** Click here for file
